# Gene Expression Changes During the Allo-/Deallopolyploidization Process of *Brassica napus*

**DOI:** 10.3389/fgene.2019.01279

**Published:** 2019-12-19

**Authors:** Qi Pan, Bin Zhu, Dawei Zhang, Chaobo Tong, Xianhong Ge, Shengyi Liu, Zaiyun Li

**Affiliations:** ^1^ National Key Laboratory of Crop Genetic Improvement, Key Laboratory of Rapeseed Genetics and Breeding of Agriculture Ministry of China, College of Plant Science and Technology, Huazhong Agricultural University, Wuhan, China; ^2^ Key Laboratory of Biology and Genetic Improvement of Oil Crops, Ministry of Agriculture, Wuhan, China; ^3^ Oil Crops Research Institute of the Chinese Academy of Agricultural Sciences, Wuhan, China

**Keywords:** *Brassica napus*, *B. rapa*, polyploidization, gene expression, DNA methylation

## Abstract

Gene expression changes due to allopolyploidization have been extensively studied in plants over the past few decades. Nearly all these studies focused on comparing the changes before and after genome merger. In this study, we used the uniquely restituted *Brassica rapa* (RBR, A_e_A_e_, 2n = 20) obtained from *Brassica napus* (A_n_A_n_C_n_C_n_, 2n = 38) to analyze the gene expression changes and its potential mechanism during the process of allo-/deallopolyploidization. RNA-seq-based transcriptome profiling identified a large number of differentially expressed genes (DEGs) between RBR and natural *B. rapa* (A_r_A_r_), suggesting potential effects of allopolyploidization/domestication of AA component of *B. napus* at the tetrapolyploid level. Meanwhile, it was revealed that up to 20% of gene expressions were immediately altered when compared with those in the A_n_-subgenome. Interestingly, one fifth of these changes are in fact indicative of the recovery of antecedent gene expression alternations occurring since the origin of *B. napus* and showed association with homoeologous expression bias between A_n_ and C_n_ subgenomes. Enrichment of distinct gene ontology (GO) categories of the above sets of genes further indicated potential functional cooperation of the A_n_ and C_n_ subgenome of *B. napus*. Whole genome methylation analysis revealed a small number of DEGs were identified in the differentially methylated regions.

## Introduction

Polyploidy (whole-genome duplication) is an important speciation mechanism for all eukaryotes, especially in higher plant evolution. Allopolyploid speciation results from interspecific hybridization and genome doubling. During the formation and evolution of allopolyploids, genome-wide genetic, epigenetic, and gene expression changes might occur, when compared with the parents (Chen, 2007; Jackson and Chen, 2010; Madlung and Wendel, 2013). On one hand, these alternations undoubtedly promote new traits formation, thereby increasing adaptability. On the other hand, heterozygosity and intergenomic interactions in allopolyploids may also result in heterosis, giving rise to phenotypic variations and increased growth vigor (Comai, 2005; Chen, 2010).

It is now realized that the genome of the allopolyploid is not just the sum of the two parental genomes. Rapid and extensive genomic changes have been revealed in the past decades in various synthetic allopolyploids or newly-formed natural polyploids (Madlung and Wendel, 2013), including sequence loss (Shaked et al., 2001; Han et al., 2005; Ozkan and Feldman, 2009; Tate et al., 2009a), gene conversion (Kovarik et al., 2008; Salmon et al., 2010), rDNA loci changes (Joly et al., 2004; Pontes et al., 2004; Lim et al., 2008), activation of transposons (Kashkush et al., 2003; Madlung et al., 2005; Chen et al., 2008; Khasdan et al., 2010; Parisod et al., 2010; Hegarty et al., 2011), and recombination between homoeologous chromosomes (Gaeta and Chris Pires, 2010; Salmon et al., 2010; Szadkowski et al., 2010). Specifically, studies have provided strong evidence that homoeologous recombination contributes much to genetic changes and might be an important driver of the genome evolution of *B. napus* (Gaeta and Chris Pires, 2010; Xiong and Pires, 2011) and coffee (Lashermes et al., 2014). Associated with these genomic changes, variations in DNA methylation between parental species and allopolyploids has also been reported in *Arabidopsis* (Madlung et al., 2005; Beaulieu et al., 2009), wheat (Zhao et al., 2011), *Brassica* (Lukens et al., 2006; Xu et al., 2009; Ksiazczyk et al., 2011), dandelion (Verhoeven et al., 2010), and many other species.

Genetic and epigenetic changes will lead to new expression patterns of duplicated genes in polyploids that might be different from those in each parental genome. The total expression level of the duplicated genes can show deviations from parental additivity (the value of the gene in the polyploids is equal to the mid-parent value, MPV). Such nonadditive gene expression patterns have been widely studied in the past in various species and are key factors determining the formation and evolution of allopolyploids (Yoo et al., 2014). For example, using microarrays, it was found that the total expression of duplicated genes in allopolyploid cotton may be similar to that of one of its parents, or may be lower or higher than those of both parents (Rapp et al., 2009; Flagel and Wendel, 2010). The first scenario was then termed as expression level dominance, which was also found in many other species (Grover et al., 2012). By directly comparing the contribution of homeologs of two parents with the transcripts pool of allopolyploids, it was found that duplicate gene pairs may express unequally, and display homeolog expression bias that usually varies among tissues and was associated with progenitor diploid expression levels (Flagel et al., 2008; Chaudhary et al., 2009; Buggs et al., 2011; Dong and Adams, 2011; Combes et al., 2013). In addition to the genetic and epigenetic changes, nonadditive gene expression could also result from many other underlying causal factors, such as maternal–paternal influence (Gong et al., 2012), gene dosage balance (Birchler and Veitia, 2010), and *cis*- and *trans* regulation (Shi et al., 2012).

However, the knowledge of genetic remodeling and gene expression changes of polyploid formation is largely derived from the studies of resynthesized polyploids that mimic natural counterparts, obtained from hybridization between the limited genotypes of extant presumed diploids in several generations. These synthetics or neo-allopolyploids commonly display phenotypic and genetic instability, low fertility, and poor seed yields. These genetic changes and resulting genome instability were possibly common during the formation of existing polyploids, which were eliminated during thousands of years of natural and artificial selection favoring stability and fertility. As natural polyploidy species formed over widely different timeframes (thousands to millions of years), except those in *Tragopogon* (Ownbey, 1950), *Spartina* (Ainouche et al., 2004), and *Senecio* (Ashton and Abbott, 1992) within the last 150 years, it is generally impossible to find their precise diploid progenitors and to accurately determine the genetic changes and interactions of parental genomes during the evolutionary process. The extant presumed diploids most closely related to the original diploid progenitors also evolved in parallel with the polyploids under the same or different environments. Also, recurrent or multiple origins for a significant proportion of polyploids (Soltis and Soltis, 2009; Tate et al., 2009b) further complicates the precise detection of genetic changes for each parental genome in natural or domesticated polyploidy species. However, the extraction of the AABB component from bread wheat (AABBDD) and the investigation of the modifications in its phenotype, karyotype, and gene expression provide novel clues for exploring the evolutionary history events after wheat formation (Kerber, 1964; Zhang et al., 2014).

*B. napus* (genomes AACC, 2*n =* 4× = 38), canola or oilseed rape in the mustard family, is the most important edible oil crop. This young species (emerging ~7,500 years ago), without known wild populations, most likely originated from multiple spontaneous and independent interspecific hybridizations between turnip rape (*B. rapa*; AA, 2*n =* 2× = 20) and cabbage or kale (*B. oleracea*; CC, 2*n =* 2× = 18) growing side by side in southern Europe or the Mediterranean region (Prakash and Hinata, 1980; Kimber and McGregor, 1995; Pratap et al., 2007). In contrast to the resynthesized *B. napus*, which shows genetic and epigenetic changes, extensive chromosomal rearrangements, and changes in gene expression and phenotype (Song et al., 1995; Pires et al., 2004; Lukens et al., 2006; Gaeta et al., 2007; Cifuentes et al., 2010; Gaeta and Chris Pires, 2010; Szadkowski et al., 2010; Xiong et al., 2011), comparative mapping and the whole gene sequence revealed that naturally domesticated *B. napus* is essentially the sum of their diploid progenitors with few genomic rearrangements (Parkin et al., 1995; Axelsson et al., 2000; Rana et al., 2004; Cheung et al., 2009; Wang et al., 2011; Chalhoub et al., 2014; Liu et al., 2014; Parkin et al., 2014). However, without access to exact parental lines, it is still unclear what changes occurred in the expression of parental genes, what factors cause these changes, and what roles these changes play in the evolution and domestication of *B. napus*.

In previous studies, we first isolated a *B. rapa* type plant from the *B. napus* “Oro” variety (Tu et al., 2010). The process of isolation included wide hybridization between *B. napus* and *Iastis indigotica* followed by continuous backcrosses between the hybrid with AAC chromosome complements (2n = 29) and *I. indigotica*. This process might include subsequent genetic events during hybrid embryo development and the growth of young plants, such as (1) the rapid elimination of paternal chromosomes; (2) chromosome doubling of the hybrid genome; and, (3) genome-specific retention or loss of maternal chromosomes. Despite the exact process, C genome chromosomes were lost step-wise during the backcrosses. Using a similar method, another restituted *B. rapa* (designated as RBR, A_e_A_e_, 2n = 20) containing the A_n_ sub-genome of *B. napus* ZS11 (A_n_A_n_C_n_C_n_, 2n = 38) was also developed (Zhu et al., 2016). Step-wise cytological investigations have revealed the complete retained A genome chromosome and the lost C genome chromosome (Zhu et al., 2016). RBR manifested many significant phenotypes at multiple growth and developmental stages that differed from those of their parental *B. napus* lines, such as small leaf lobes, short siliques, and greater susceptibility to diseases during and after flowering in humid and high-temperature environments. Particularly, as with many other *B. rapa* lines, RBR showed severe self-incompatibility that resulted in poor seed set by self-pollination, although with a high pollen stainability (Zhu et al., 2016). Because its parent ZS11 is self-compatible, this result indicated that the self-incompatibility of RBR might result from the loss of C genome interaction when the A genome was extracted. The availability RBR and representative natural *B. rapa* and *B. oleracea* provide a unique opportunity to investigate the gene expression changes of the A genome during the extraction process or those that occurred over time at the allotetraploid level. Here, we detail these changes and provide a potential mechanism.

## Materials and Methods

### Plant Materials and Nomenclature of Different Genomes

A *B. rapa* line (designated as RBR, restituted *B. rapa*) with an “extracted” genome (A_e_A_e_) of the allotetraploid *B. napus* (cultivar Zhongshuang 11; designated as ZS11) was produced previously (Zhu et al., 2016). The A and C subgenome within nucleus of *B. napus* was named as A_n_ and C_n_, respectively. RBR was propagated *via* self-pollination for one more generation after its extraction and the second generation was used in this study. *B. napus* ZS11, four varieties of *B. rapa*, one belonging to var. *pekinensis* (cultivar Chiifu-401) and three belonging to var. *campestris* (TRA1, RA2 and BY1) and three varieties of *B. oleracea* belonging to var. *capitata* (Ganlan cv02-12), var. *alboglabra* (Cjielan) and wild type (TRC) were also used in this study (**Supplementary Table 1**). The A genome within nucleus of four natural *B. rapa* was named as A_r_. The relationships of all materials used in this study were descripted in **Supplementary Figure 1**. All plants were grown in a greenhouse under controlled conditions at a 22/20°C day/night cycle, with 16-h day length. For DNA and RNA extraction, young leaves were collected at four-leaf development stages.

### RNA Extraction and Transcriptome Analysis

Total RNA was extracted from the young leaves of ZS11, RBR, four *B. rapa* varieties and three varieties of *B. oleracea* using Trizol reagent (Invitrogen, CA, USA) and purified using RNeasy Mini Spin Columns (Qiagen, Germany). For each variety used in this study, RNA was extracted independently at least from two plants (**Supplementary Table 1**). The integrity of RNA was checked with the Agilent Bioanalyzer 2100 Eukaryote Total RNA Nano Series II system. Individual mRNA-Seq libraries (Strand-Specific) with insert sizes of 300 bp for each sample were constructed and sequenced with paired-end reads of 150 bp on the Illumina Hiseq 4000 platform. FastQC (http://www.bioinformatics.babraham.ac.uk/projects/fastqc/) and the NGSQC Toolkit (v2.3) (Patel and Jain, 2012) was used to check, visualize, and control the quality of RNA-seq reads.

To make comparisons of gene expression across species, all clean mRNA-Seq reads were then mapped to the *B. napus* ‘Darmor-*Bzh*' reference genome using BWA (Li and Durbin, 2009) with default parameters (Version: 0.7.8-r455), and the uniquely mapped reads were used for further analysis. Differentially expressed genes or homoeologous gene pairs were analyzed using the DESeq package (Anders and Huber, 2010). The raw read counts for each gene were calculated using HTSeq-count (v0.6.1) (Anders et al., 2015) under union mode and were normalized by respective size factors, which were estimated with the function estimateSizeFactors in the DESeq package. Two-fold changes and *P* values <0.05 were characterized as DEGs according to a negative binomial model of DESeq and multi-factor analyses relying on the generalized linear model (GLM) formulation.

### DNA Methylation Analysis Based on Bisulfite-Treated DNA Sequencing

Qualified genomic DNA of ZS11 and RBR, with two biological duplicates (**Supplementary Table 1**), along with 0.5% w/w of unmethylated lambda DNA (Promega, Madison, WI, US) included to assess bisulfite conversion efficiency, was sheared using the Covaris S220 sonicator to produce an average fragment size of 200–300 bp. Library construction was performed using the Illumina Paired-End Sample Prep Kit and QIAGEN PCR purification kits according to the Illumina whole-genome bisulfite sequencing for methylation analysis protocol. Methylated adapters from the Illumina TruSeq DNA Sample Prep Kit v2 were substituted for the unmethylated adapters within the Illumina Paired-End Sample Prep Kit. Ligation products equivalent to 450 bp were purified using the BluePippin DNA size selection system (Sage Science). Bisulfite conversion of this DNA was performed using the EZ DNA Methylation Gold Kit (Zymo Research). The libraries were quantified using a BioAnalyzer (Agilent) and a KAPA library quantification kit for Illumina (KAPA Biosystems) and sequenced from both ends (paired-end) for 100 cycles on either a 2,000 or 2,500 platform, and 125 bp paired-end reads were generated.

Trimmomatic (version 0.32) (Bolger et al., 2014) was used for filtering adapters and low-quality reads for raw bisulfite-treated DNA sequencing data. Bismark software (Krueger and Andrews, 2011) was used to align the clean reads to the reference genome using the default parameters. The methylation level for each C cytosine was calculated using the Bismark methylation extractor based on uniquely mapped reads. Fisher's exact test was used for the identification of differentially methylated regions (DMRs) with a false discovery rate (FDR) < 0.01 in 200 bp windows of the *B. napus* genome.

### Gene Ontology (Go) Enrichment Analysis

GO annotations were performed using agriGO (http://bioinfo.cau.edu.cn/agriGO/index.php). The Singular Enrichment Analysis tool was used to do the GO annotations and significant GO term enrichment analysis (Du et al., 2010), which computed GO term enrichment in one set of genes by comparing it with another set, named the target and reference list, respectively. Enrichment was calculated by Fisher's exact test with Hochberg's multitest adjustment (FDR, P <0.05).

## Results

### Transcriptome Changes in the A_n_ Subgenome of *B. napus* Reflect Effects of Allohexaploidization/Domestication

Because we do not know the exact original progenitor *B. rapa* of ZS11, we compared the transcriptome of A_n_-subgenome and RBR with those of natural *B. rapa* (A_r_A_r_, 2n = 20), which represented by four cultivars of two subspecies, *pekinensis* (Chiifu-401) and *campestris* (TRA1,TRA2, BY1) using RNA-seq (**Supplementary Figure 1**). We found 15,614(47.3%), 11,642(36.9%), 13,574(42.7%) and 6,218(19.3%) genes to be differentially expressed in A_n_-subgenome compared to those in Chiifu-401, TRA1, TRA2 and BY1, respectively (**Supplementary Table 2**). Similarly, there were 14,184 (43.8%), 10,366 (33.7%), 13,034 (43.9%), and 3,142 (10.0%) DEGs between RBR and each *B. rapa*, respectively (**Supplementary Table 3**). Obviously, transcriptome divergence between A_n_-subgenome of ZS11 and BY1 is the least and the divergence between A_n_-subgenome and Chiifu-401 is the greatest. The same is true with RBR. These results were consistent with the known breeding history of ZS11 and the evolution and/or domestication histories of these *B. rapa* lines: (1) BY1 has been used as one of the cross parents during the breeding of ZS11 (**Supplementary Figure 1**); (2) Chiifu-401 is domesticated for use as a vegetable, whereas the other *B. rapa* are used as oilseeds.

**Figure 1:**
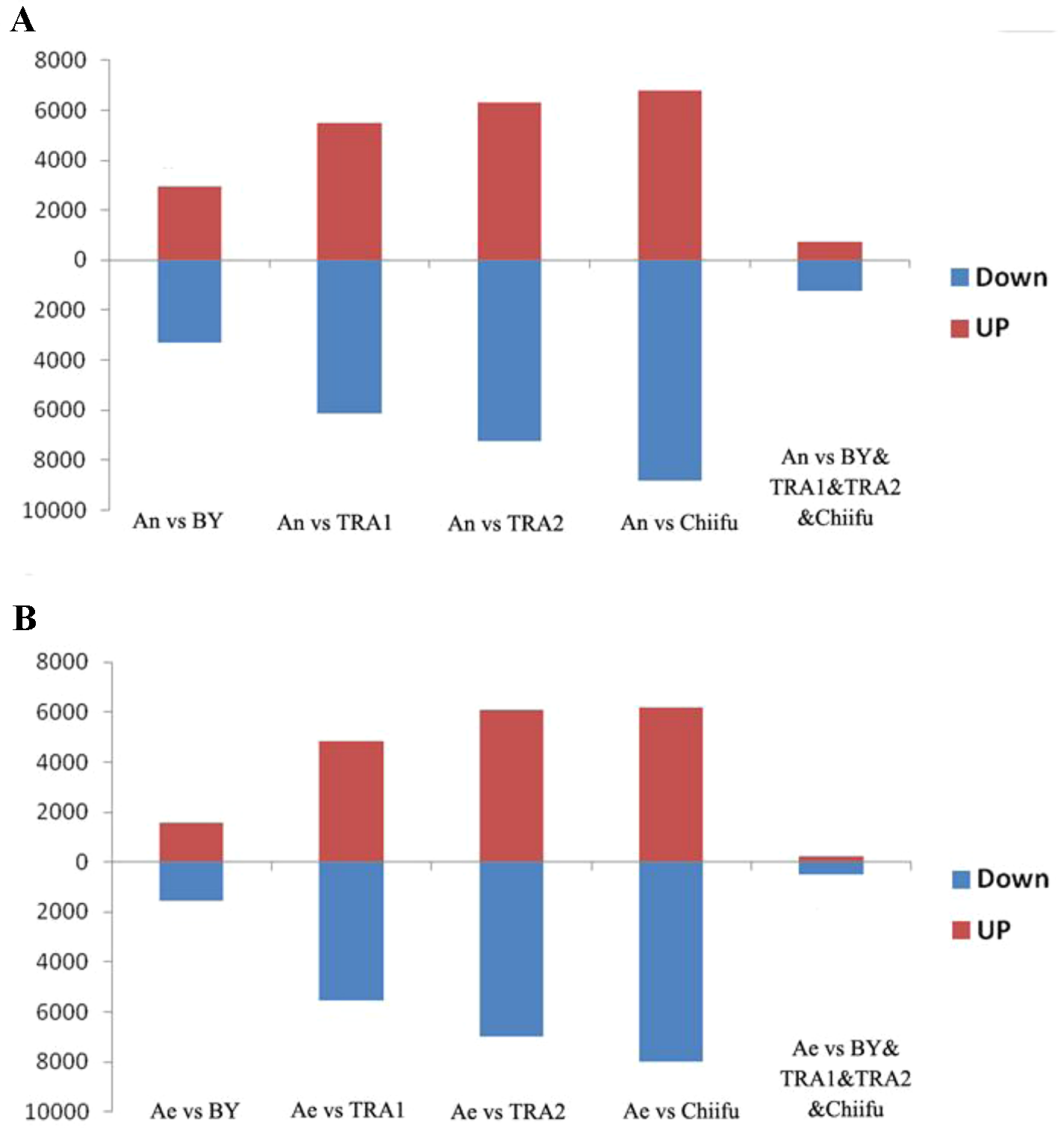
Histograms of the up- and downregulated gene numbers in each of the four pairwise comparisons between A_n_-subgenome **(A)** and RBR **(B)** and four varieties of *B. rapa*: Chiifu-401, BY1, TRA1, and TRA2. An represented A_n_-subgenome of *B. napus* ZS11 and Ae represented RBR.

Next, we analyzed the proportions of upregulated versus downregulated genes in paired comparisons. When comparing A_n_-subgenome with each *B. rapa*, BY1, TRA1, TRA2 and Chiifu-401, 3,281(52.8%), 6,139(52.7%), 7,259(53.5%) and 8,803(56.4%) DEGs, respectively, were found to be upregulated, whereas 2,937(47.2%), 5,503(47.3%), 6,315(46.5%) and 6,811(43.6%) DEGs, respectively, were found to be downregulated (**Supplementary Table 2**; **Figure 1A**). When comparing RBR with each *B. rapa*, 1,573 (50.1%), 5,542 (53.5%), 6,964 (53.4%), and 7,997 (56.4%) DEGs, respectively, were found to be upregulated, whereas 1,569 (49.9%), 4,824 (46.5%), 6,070 (46.6%), and 6,187(43.6%) DEGs, respectively, were found to be downregulated (**Supplementary Table 3**; **Figure 1B**). For each comparison, the numbers of upregulated genes were significantly higher than the numbers of downregulated genes in A_n_-subgenome and RBR, except for that in the comparison between RBR and BY1, according to a binomial test (**Supplementary Tables 2** and **3**). These results indicated that the gene expression in A_n_-subgenome of *B. napus* and A_e_ of RBR changed towards a generally higher level than those in A_r_ genome of natural *B. rapa*. Notably, this trend was most pronounced between A_n_-subgenome/RBR and Chiifu-401, indicating the combined effects of allopolyploidization and selection under domestication.

To test this speculation further, we analyzed shared DEGs between A_n_-subgenome and four natural *B. rapa* as well as between RBR and four natural *B. rapa*. We found that 1,247 common genes were upregulated and 740 genes were downregulated in the A_n_-subgenome of ZS11 (**Supplementary Table 2**; **Figure 1A**). We also found that 507 common genes were upregulated and 242 genes were downregulated in RBR (**Supplementary Table 3**; **Figure 1B**). In both cases, the numbers of upregulated genes were significantly higher than those of downregulated genes. These changes may be considered as the results of the effects of allopolyploidization/domestication occurring since the origin of *B. napus*. Together, these transcriptome comparisons suggest that allopolyploidization and domestication at the tetraploid level might have significantly increased the expression level of a substantial proportion of genes in the A_n_-subgenome of *B. napus* relative to its progenitor *B. rapa*.

### Reversible Gene Expression Changes During the Allo-/Deallopolyploidization of *B. napus*

**Figure 2:**
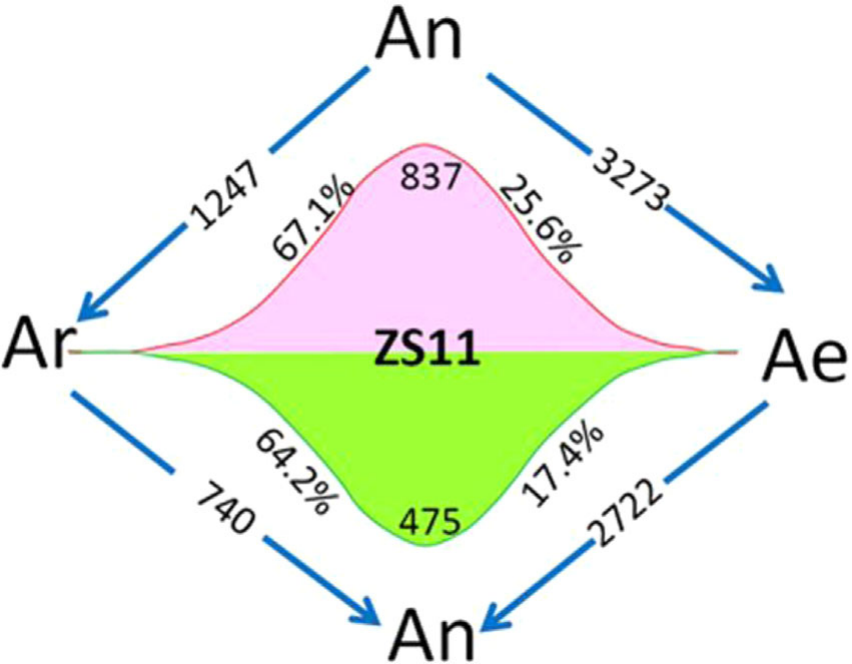
Diagram of the total number of genes specially up- and downregulated in the A subgenome of ZS11 (A_n_) in comparison with RBR (A_e_) and four natural *B. rapa* lines (A_r_). The arrows indicate the genomes in which genes are down-regulated, and the numbers inside represent the number of downregulated genes. Red and green wavy lines indicate a continuous trend of gene expression from A_r_ to A_n_ and A_e_. The numbers represent the numbers of genes with this expression trend, and the percentages represent the proportions of these genes in the total up- and downregulated genes in each pairwise comparison. A_r_ vs A_n_ represented DEGs shared by all *B. rapa* varieties versus A_n_-subgenome of *B. napus.* A_r_ vs A_e_ represented DEGs shared by all four *B. rapa* versus RBR. A_n_ vs A_e_ represented DEGs between RBR and those in A_n_-subgenome of *B. napus* ZS11.

Using four natural *B. rapa* representing the original diploid progenitor of *B. napus,* together with RBR, we were able to analyze the reversible gene expression changes occurring during the allo-/deallopolyploidization process in *B. napus*. We compared the transcriptome of RBR with that of *B. napus* A_n_ subgenome by RNA-seq analysis with three biological replicates. We found that 5,995 genes (18.8% of a total of 31,867 genes) were differentially expressed between A_e_ of RBR and A_n_ subgenome of ZS11. Among these genes, 3,273 (53.7%) were downregulated and 2,722 genes were upregulated in RBR. The numbers of down regulated genes were significantly higher than that of the upregulated genes (**Supplementary Table 3**). In order to investigate whether these changes are in fact indicative of the recovery of antecedent changes occurring during the formation and evolution of *B. napus*, we analyzed the expression of shared DEGs between A_n_-subgenome and four natural *B. rapa* in RBR. We found that 837 (67.1%) of 1,247 upregulated genes and 475 (64.3%) of 740 downregulated genes in A_n_ subgenome of ZS11 were down- and up-regulated in RBR, respectively. These genes represented 25.6% of the total 3,273 downregulated genes and 17.4% of the total 2722 upregulated genes observed during *B. rapa* extraction (**Figure 2**). Obviously, by the unique restitution of *B. rapa* RBR, expression changes of a set of genes (21.5%) were revealed to be reversible during the allo-/deallopolyploidization of *B. napus*. In other words, these genes are highly or lowly expressed specifically in the A genome at the tetraploid level in *B. napus*. So, it is reasonable that the pattern of gene expression of the A genome in RBR was changed rapidly towards that in the natural *B. rapa* after extraction from the nucleus of *B. napus*.

### *B. napus-*Specific Upregulated Genes Are Enriched in Distinct Gene Ontology Categories

**Figure 3:**
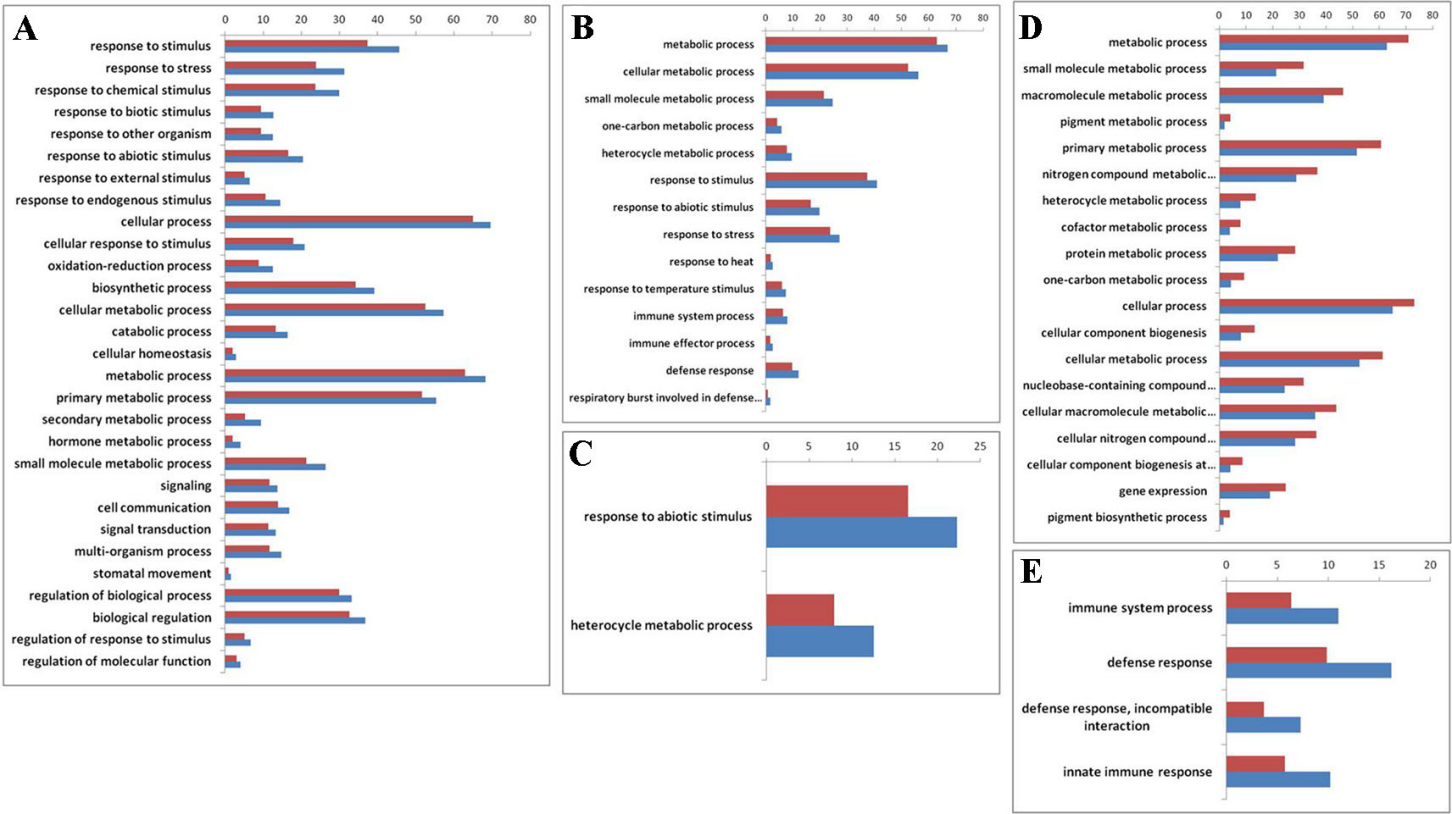
Gene ontology (GO) enrichment (> 50%) for genes that are differentially expressed before and after RBR extraction, specially up- and downregulated in *B. napus* as well as between natural *B. rapa* and *B. oleracea*. The x-axis is the GO category terms, and the y-axis is the percentages of genes mapped by the GO category terms, which are calculated by the number of genes mapped to a given GO category divided by the number of all mapped genes (Vermilion bars denote the percentages of each GO category in all expressed genes, 26,539 in total). **(A)** Gene ontology analysis of the 2722 upregulated genes in RBR comparing to those in An-subgenome of B. napus. **(B)** Gene ontology analysis of the 3,273 downregulated genes in RBR comparing to those in An-subgenome of *B. napus*. **(C)** Gene ontology analysis of the upregulated 837 genes in An-subgenome of *B. napus* comparing to those in natural *B. rapa* as well as in RBR. **(D)** Gene ontology analysis of the upregulated 455 homoeologous genes in *B. rapa* comparing to those in *B. oleracea*. **(E)** Gene ontology analysis of the 518 upregulated homoeologous genes in *B. oleracea* comparing to those in *B. rapa.* Note: only the Ontology of biological process was shown here for each enrichment.

To explore whether those genes with reversible expression patterns in the allo-/deallopolyploidization are related to specific functional features, we performed a gene ontology (GO) analysis. We found that both down- and upregulated genes in RBR versus those in the A_n_ subgenome of *B. napus* showed over-representation of several categories of genes involved in metabolism and responses to stress stimulus (**Supplementary Datasheet 2**; **Figure 3** **A**, **B**). Particularly, *B. napus-*specific upregulated genes, either compared with natural *B. rapa* or with RBR, showed over-representation for two categories: heterocyclic metabolic processes and responses to abiotic stimuli (**Supplementary Datasheet 1**; **Figure 3** **C**). In order to elucidate the origins of these changes of A_n_-subgenome of *B. napus*, we compared the homoeologous gene (**Supplementary Datasheet 1**) expression in two parental species of *B. napus: B. oleracea* and *B. rapa*, which were represented by four varieties of *B. rapa* and three varieties of *B. oleracea*. In total, 12 pairs of comparisons were made (**Supplementary Table 4**). Among the DEGs in each comparison, 455 and 518 upregulated genes were shared by all *B. rapa* and all *B. oleracea* lines, respectively. GO enrichment analysis found that 455 highly expressed genes in *B. rapa* showed several enriched GO categories involved in metabolic processes, whereas 518 highly expressed genes in *B. oleracea* lines showed several enriched GO categories involved in stress responses (**Supplementary Datasheet 2**; **Figure 3** **D**, **E**). These results indicate that there are differences in capabilities of metabolism and disease resistance between natural *B. rapa* and *B. oleracea*. A_n_ subgenome-specific up-/downregulated genes shown enrichment in metabolism and responses to stress might result from *trans* regulation, either positive or negative, by the C_n_ subgenome.

### Immediate and Dramatic Gene Expression Changes of A_n_ Subgenome During Its Extraction Are Associated With A_n_ and C_n_ Homoeologous Expression Bias

Studies in *B. napu*s lines showed that no pronounced genome dominance was present, but there are significantly biased contributions to the gene expression of the A_n_ and C_n_ homeologs in different tissues (Chalhoub et al., 2014). We also analyzed the contribution of homeolog A_n_ and C_n_ (Chalhoub et al., 2014) to the transcriptome of *B. napus* in young leaves used here. ZS11 showed significantly higher expression for 3,323 (12.9%) A_n_ homeologs and 3,542 (13.7%) C_n_ homeologs in 25,699 total gene pairs. There are 18,834 (73.3%) gene pairs in ZS11, which showed equal contributions to the transcriptome. These results are consistent with those found in Darmor-*bzh* (Chalhoub et al., 2014).

**Figure 4:**
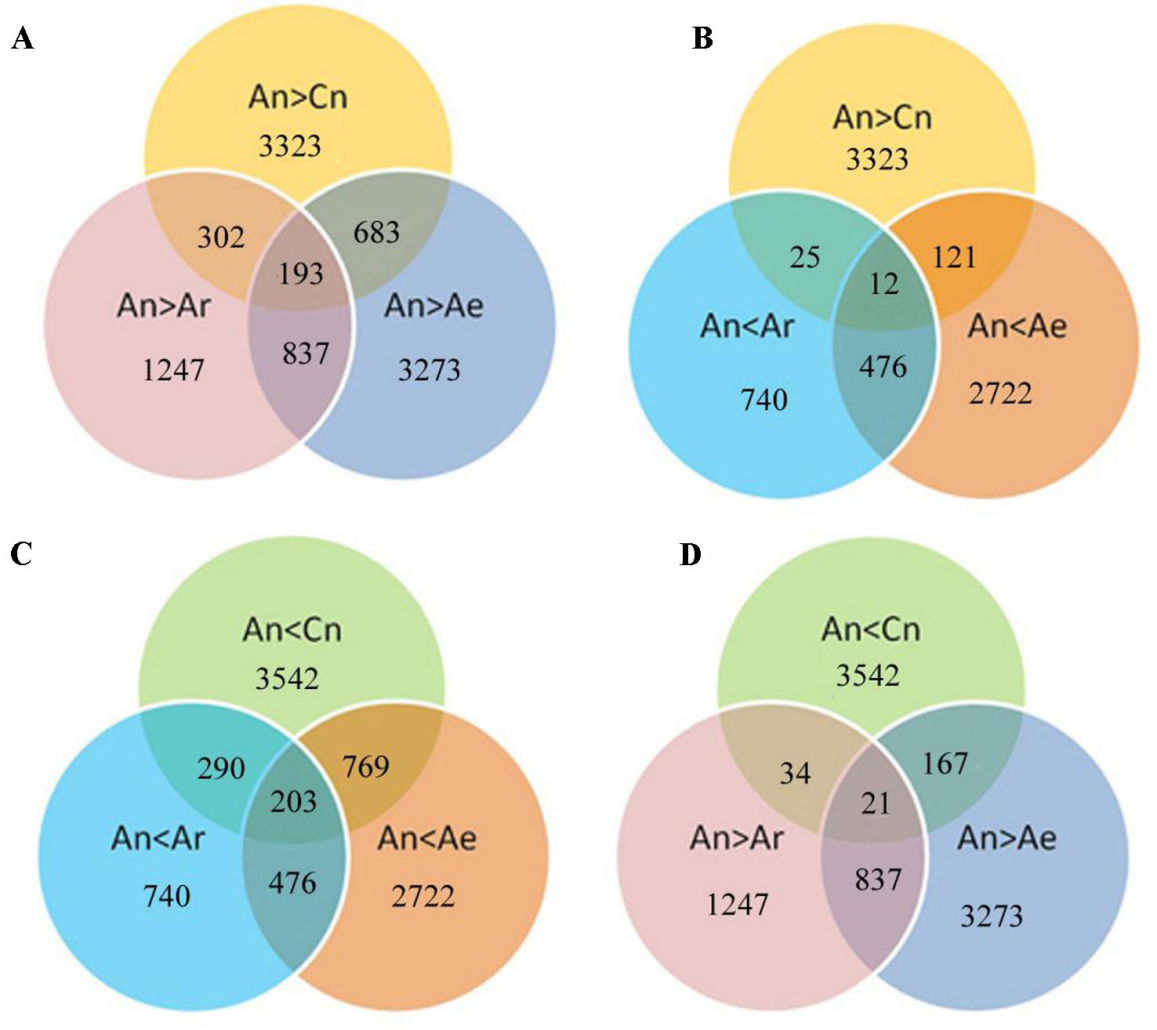
Diagram shows the potential relationships between gene expression changes in pairwise comparisons (A_e_ vs A_n_ and A_n_ vs A_r_) and homoeologous expression bias of A_n_ and C_n_. A, B: Specially up- **(A)** and downregulated **(B)** genes under A_n_ > C_n_; C, D: Specially up **(C)** and down **(D)** regulated genes under A_n_ < C_n_.

We next analyzed the expression of these differentially expressed gene pairs in RBR and natural *B. rapa*. A total of 683 (20.5%) and 302 (9.1%) of the 3,323 highly expressed A_n_ homeologs in *B. napus* ZS11 were downregulated in RBR and in all natural *B. rapa*, respectively (**Figure 4A**). Moreover, 193 common genes were identified simultaneously in gene sets of A_n_ > C_n_, A_n_ > A_r_ and A_n_ > A_e_ (**Figure 4A**). In contrast, only 121, and 25 of 3,323 highly expressed A_n_ homeologs were upregulated in RBR and all natural *B. rapa*, respectively. Only 12 common genes were found simultaneously in above three gene sets (**Figure 4B**). Similarly, 769 (21.7%) and 290 (8.2%) of 3,542 A_n_ homeologs with lower expression were upregulated in RBR and all natural *B. rapa*, respectively, and 203 common genes were identified simultaneously in gene sets of A_n_ < C_n_, A_n_ < A_r_ and A_n_ < A_e_ (**Figure 4C**). However, only 167 and 34 genes were downregulated in RBR and four natural *B. rapa*, respectively, and 21 common genes were found in gene sets of A_n_ < C_n_, A_n_ < A_r_ and A_n_ < A_e_ (**Figure 4D**). Taken together, these results indicated that the A_n_ homeologs that showed higher expression than C_n_ homeologs in *B. napus* tended to be downregulated in extracted *B. rapa* and natural *B. rapa*, while the A_n_ homeologs with lower expression tended to be upregulated.

### Alternations in DNA Methylation in AA Components After Its Extraction From *B. napus*

**Figure 5:**
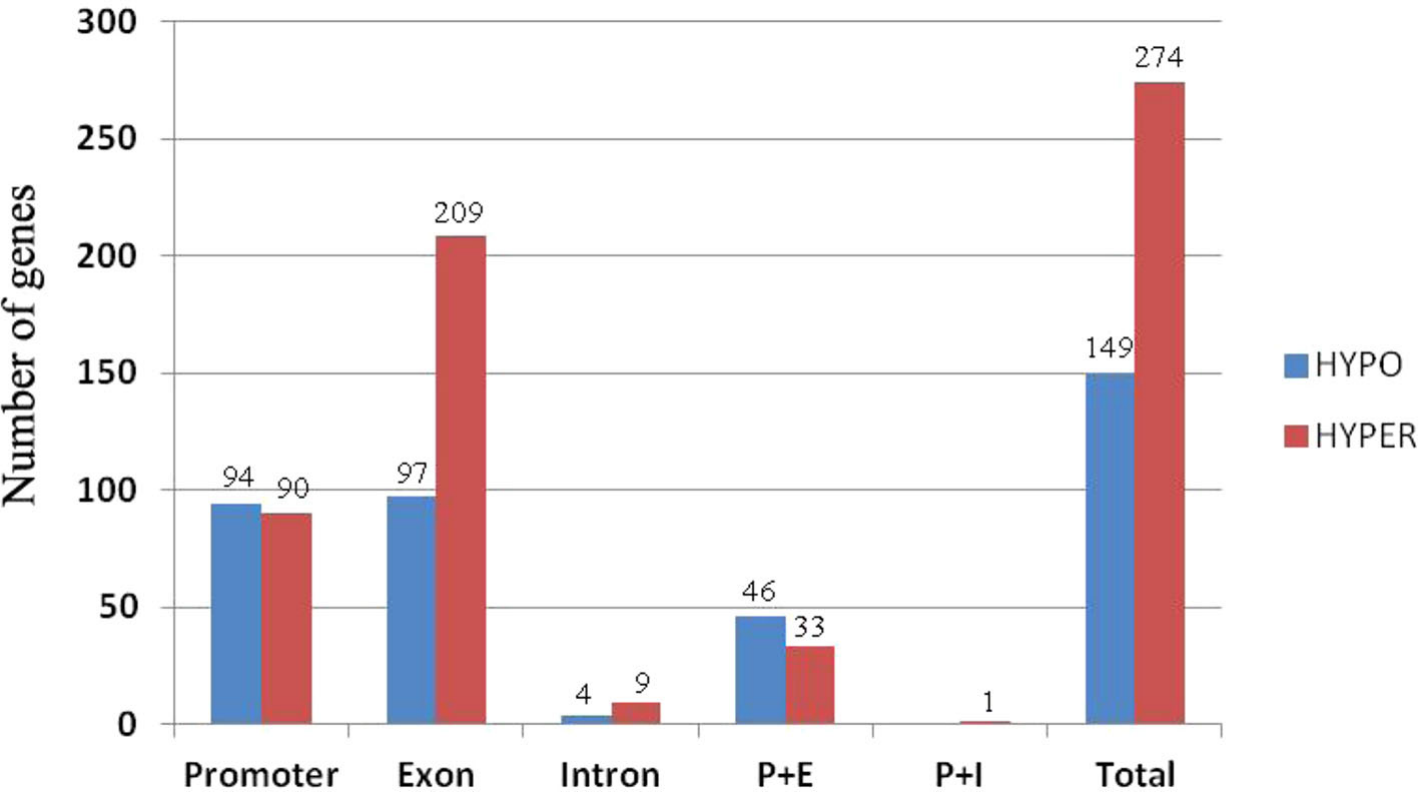
Histograms of the numbers of genes with alternations in methylation status at different locations. P+E represents methylation changes involved in promoter and exon regions and P+I represent methylation changes involved in promoter and intron regions.

To investigate how DNA methylation changes occurred in RBR immediately after its extraction from *B. napus,* which might contribute to the gene expression changes, we performed whole genome bisulfite sequencing analysis of RBR, as well as of ZS11. For RBR and ZS11, 59,473,375 and 106,938,717 raw reads were produced, respectively. After removal of those low-quality data, 57,134,049 and 102,602,342 clean reads were produced, respectively. Using the *B. napus* genome as a reference, 30,586,180 (53.53%) and 46,804,759 (45.62%) reads were uniquely mapped. In total, 43.21%, 18.34%, and 3.96% of the total CG, CHG, and CHH (where H = A, C or T) loci were methylated in RBR, and 55.50%, 27.90%, and 5.81% of the total loci were methylated in ZS11. The overall methylation at different loci in the later is consistent with those in sequenced *B. napus* leaves, in which 53% CG, 22% CHG, and 7% CHH were methylated (Chalhoub et al., 2014). To compare methylation levels between RBR and the A_n_-subgenome within *B. napus*, the DMR (differentially methylated region) for each was identified. In total, there are 1,523 (500 bp-4.5 kb) and 4,654 (500 bp-4.7 kb) regions, which span about 0.98 Mb and 3.07 Mb of the genome, respectively, show hypo- and hyper-methylation in RBR versus ZS11 (**Supplementary Datasheet 3**). Obviously, RBR showed higher numbers of hyper-methylated regions than that of hypo-methylated regions immediately after its extraction. We found 274 (6.4% of total 3,726) genes were down-regulated in RBR exactly within the hyper-methylated regions, while 149 (4.7% of total 3,204) upregulated genes were located in hypo-methylated regions. In both cases, most of the DEGs within DMR have altered methylation involved in promoter and exon regions (**Figure 5**). Interestingly, there are many more genes in hyper-methylated regions (207) than in hypo-methylated regions (93), with changed methylation status involved in exon (**Figure 5**). Overall, more regions were changed toward hyper-methylation in RBR, which is consistent with the prevalence of more downregulated genes than upregulated genes in RBR compared to those in A_n_ subgenome of *B. napus*. GO enrichment analysis revealed that 274 down-regulated genes within hyper-methylated regions in RBR involved biological process mainly in meiotic chromosome organization and in molecular function in calcium ion binding, DNA binding, transferase activity, nucleic acid binding and methyltransferase activity. Meanwhile, 149 upregulated genes located in hypo-methylated regions showed enrichment in biological of disaccharide metabolic process, oligosaccharide metabolic process, hormone biosynthetic and metabolic process, etc. and molecular function of lyase activity, phosphoric ester hydrolase activity and phosphatase activity (**Supplementary Datasheet 4**).

## Discussion

Over the past few decades, researchers have revealed a wide range of changes in parental gene expression during the formation and evolution of allopolyploids, using different methods investigating natural and synthetic allopolyploids (Adams et al., 2003; Hegarty et al., 2006; Wang et al., 2006; Chaudhary et al., 2009; Pumphrey et al., 2009; Rapp et al., 2009; Akhunova et al., 2010; Chague et al., 2010; Buggs et al., 2011; Grover et al., 2012; Li et al., 2014). These changes occur either immediately after polyploid formation or over long periods of evolution. Although the molecular mechanisms are still not well understood, genetic and epigenetic changes of parental genomes, *cis* and *trans* regulation between different genomes, gene dosage, and stochastic effects are the likely main mediators of these changes (Wendel, 2000; Osborn et al., 2003; Riddle and Birchler, 2003; Adams and Wendel, 2005; Comai, 2005; Chen, 2007; Doyle et al., 2008; Soltis and Soltis, 2009; Jackson and Chen, 2010; Birchler, 2012; Madlung and Wendel, 2013; Yoo et al., 2014). However, gene expression changes of a given subgenome(s) within an allopolyploid species has been studied only in polyploid genome environment, thus obstructing any assessment of transcriptome changes to a specific subgenome(s) without confounding effects (e.g., trans-acting factors) from the other subgenome(s). RBR, which was obtained by a deallopolyploidization process, is thus a fascinating plant for analysis of genomic and/or gene expression changes in the A_n_-subgenome of *B. napus* accumulated since its origin. Here, without the affects from C_n_-subgenome, we found that RBR lines have significantly more upregulated genes than downregulated genes in comparison with natural *B. rapa*. This result indicated that some gene expression of A genome had been changed directionally and permanently since it merged with the C genome in the allotetraploid genome of *B. napus*. However, it should be pointed out that the exact founder genotype of *B. rapa* that donated the A_n_ sub-genome of *B. napus* ZS11 might be different from those we analyzed, an issue largely prevalent in all studies using model extant accessions to represent actual progenitors of naturally formed polyploids (Gottlieb, 2004; Zhang et al., 2014).

Gene expression changes originating from the genetic and heritable epigenetic will be “irreversible,” while changes arising from the other mechanism might be “reversible” regarding the subgenome(s) of a given allopolyploid. RBR which was extracted from *B. napus,* a neopolyploid, also provide the opportunity to identify genes with reversible expression pattern. This way, we found that 837 and 475 genes were specifically up- and downregulated in *B. napus*, respectively, suggesting that the changes of these genes are “reversible” and might be caused by *trans* factors or gene dosage effects, for example by small RNA (Palacios et al., 2019). Because these genes are common to both RBR and the A_n_ sub-genome of *B. napus* in comparison with the four natural *B. rapa*, they would be underestimated in number if the investigation was performed on the same line before polyploid formation and after the extraction. These results also indicated that genes of A_e_ genome tend to be expressed at levels as in A_r_ genome of natural *B. rapa* after its extraction from the A_n_A_n_C_n_C_n_ genome, despite coevolved with the C genome for thousands years.

Gene expression changes might result from both genetic and epigenetic mechanisms (Madlung and Wendel, 2013). Due to the close relationship between the A and C sub-genomes and the lack of genetic mechanisms suppressing homoeologous pairing (Cui et al., 2012), homoeologous exchanges (HE) is considered a very important mechanism in *B. napus* evolution and domestication, and has been widely investigated in *B. napus* in particular (Udall et al., 2005; Szadkowski et al., 2010; Xiong et al., 2011; Chalhoub et al., 2014; Stein et al., 2017; Sun et al., 2017; Hurgobin et al., 2018). Upon analysis of the DEGs in each comparison in this study, the genes involved in HE (Homoeologous exchanges) had been removed; thus, those alternations might stem from small fragment changes such as deletions, insertions, and SNPs. In resynthesized *B. napus,* initial generations showed extensive changes at the genomic level, including deletion bands and novel bands (Song et al., 1995; Lukens et al., 2006; Gaeta et al., 2007; Cui et al., 2013). These changes cannot be entirely attributed to the homoeologous changes driven by meiosis because these changes are detected at the S_0_ generation (Lukens et al., 2006; Cui et al., 2013). Accordingly, by AFLP analysis with 57 pairs of primers, it was found that RBR not only lost 34.51% of the *B. napus*-specific fragments which due to the C genome chromosome lost but also have 13.41% of fragments novel for two parents. Also, changes in DNA methylation are usually associated with changes in gene expression, and heritable epigenetic alterations may contribute (Lukens et al., 2006; Xu et al., 2009; Cui et al., 2013). In bread wheat, permanent silencing of gene homeologs can be caused by altered DNA methylation (Shitsukawa et al., 2007; Hu et al., 2013). Similarly, we found that about 423 (6.1% of total 6930) DEGs between RBR and the A_n_-subgenome exclusively occurred within DMR regions.

Our GO enrichment analysis indicated that highly expressed genes in *B. rapa* and *B. oleracea* showed several enriched GO categories involved in metabolic processes and defense responses, respectively (**Figure 3**). We showed that A_n_ subgenome-specific up-/downregulated genes shown enrichment in metabolism and responses to stress might result from *trans* regulation, either positive or negative, by the C_n_ subgenome. These results are consistent with the observation that *B. oleracea* usually shows greater disease resistance than *B. rapa* but grows more slowly. In China, *B. napus* has replaced the native *B. rapa* and *B. juncea* owing to its higher seed yield and stronger resistance to biotic and abiotic stresses. Empirically, it was considered that the improved resistance of *B. napu*s was largely contributed by C_n_ subgenome which was origin from *B. oleracea*. The diminished vigor and strong susceptibility to diseases of RBR also suggested that the functional partitioning of disease resistance was enhanced for the C subgenome but attenuated for the A subgenome in *B. napus*. Based on the above results, we suggest that during *B. napus* formation and evolution, genes from *B. rapa* related to defense responses might have been transiently upregulated, whereas those associated with biosynthetic and metabolic processes were transiently downregulated by *trans* regulation from the C genome. These types of regulation also led to the differential expression between the A_n_ and C_n_ homeologs formed in *B. napus*. However, it must be pointed out that the current results are mainly based on transcriptome analysis, and experiments on specific gene expression, regulation, and function analysis is needed in future for further confirmation.

## Conclusions

Through unique restitution of *B. rapa*, we were able to investigate the gene expression changes of the AA component of *B. napus* at allotetraploid level accumulated since its formation, and revealed reversible gene expression changes that occurred during the process of allo-/deallopolyploidization. Functional analysis of involved genes indicated the potential cooperation of parental genomes, which will shed lights on subgenome-specific contribution to polyploids.

## Data Availability Statement

All raw data from this study have been submitted to the NCBI SRA database (http://www.ncbi.nlm.nih.gov/sra/) under the project number PRJNA480641.

## Author Contributions

ZL, XG, and SL conceived and designed this study. BZ prepared the materials and plants. BZ, XG, CT, QP, and DZ analyzed the data. CT, XG, ZL, and SL wrote and revised the manuscript. All authors have read and approved the final version of the manuscript.

## Funding

This research was supported by National Key Research and Development Program of China (grant 2016YFD0100202, 2018YFE0108000, 2016YFD0100305, 2016YFD0101007, 2016YFD0100602), National Natural Science Foundation of China (grant 31471530, 31801391, 31770250), Natural Science Foundation of Hubei Province (2019CFB628) and the Basic CAAS Fundation for International Cooperation (Y2019GH08).

## Conflict of Interest

The authors declare that the research was conducted in the absence of any commercial or financial relationships that could be construed as a potential conflict of interest.
