# Supplementary material for: Gene Expression Changes During the Allo-/Deallopolyploidization Process of Brassica napus
Source: Front Genet. 2019 Dec 19;10:1279. doi: 10.3389/fgene.2019.01279 (PMC6931035; doi:10.3389/fgene.2019.01279)
Supplement: Supplementary file 5 [file Image_1.pdf]

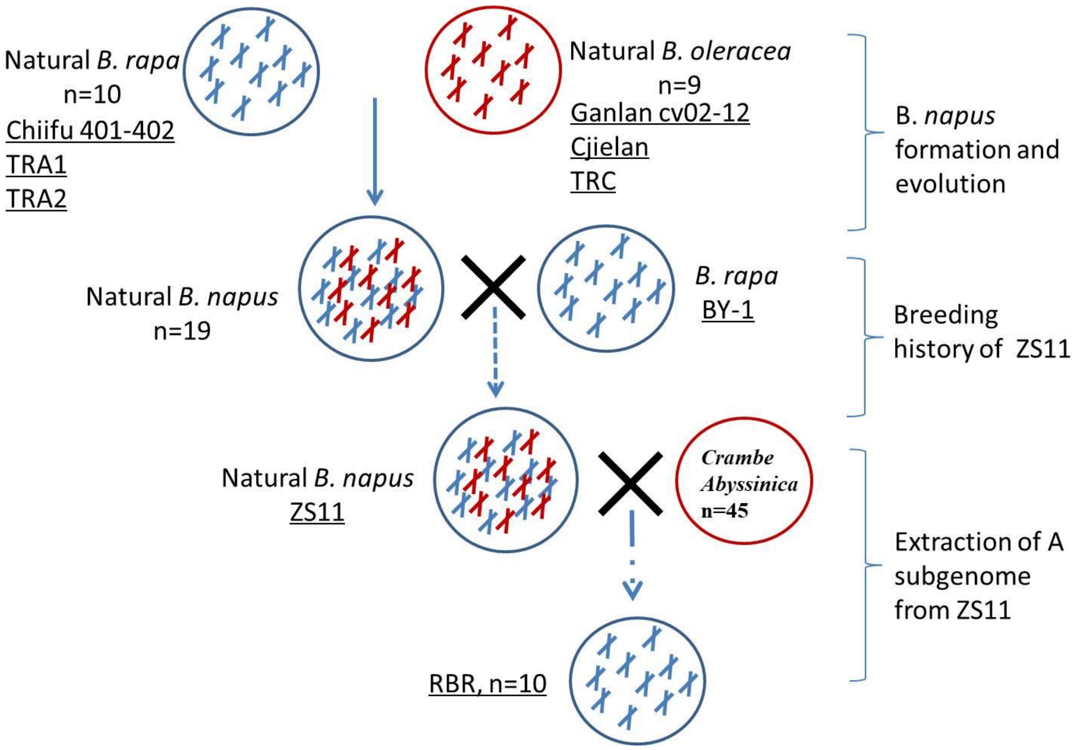

Supplementary Figure 1. The relationships of materials used in this study. The solid line arrow represents that the natural *B. napus* was evolved via hybridization between natural *B. rapa* and *B. oleracea* but the exact parents are unknown. The dotted line arrow represents that "BY-1" was used as one of parents in breeding process of ZS11. Solid and dotted line arrow represents that RBR was extracted via wide hybridization between *B. napus* and *Crabe Abyssinica.* Materials underlined are used in this study.
