# Supplementary material for: Gene Expression Changes During the Allo-/Deallopolyploidization Process of Brassica napus
Source: Front Genet. 2019 Dec 19;10:1279. doi: 10.3389/fgene.2019.01279 (PMC6931035; doi:10.3389/fgene.2019.01279)
Supplement: Supplementary file 6 [file Presentation_1.pdf]

Supplementary Table 1. Plant materials used in this study

Species	Name or accession number	Origin	RNA-seq	Bisulfite sequence
Extracted *B. rapa*	RBR	China	Y(3)	Y(2)
Natural	Chiifu 401-402	China	Y(3)	N
B. rapa	BY-1	China	Y(3)	N
	TRA1	Germany	Y(2)	N
	TRA2	Germany	Y(2)	N
B. oleracea	Ganlan cv02-12	China	Y(3)	N
	Cjielan	China	Y(2)	N
	TRC	England	Y(2)	N
B. napus	ZS11	China	Y(3)	Y(2)

Note: Y indicated that the expriments was done while N represented not; the number in brackets represented biological duplications.

Supplementary Table 2. Differentially expressed genes (DEGs) between A_n-subgenome of *B. napus* and four natural *B. rapa*

	Total pairs	Total DEGs	Up	Down	P-value
BY vs A_n	32193	6218(19.3%)	2937(47.2%)	3281(52.8%)	1.356e-05
TRA1 vs A_n	31526	11642(36.9%)	5503(47.3%)	6139(52.7%)	3.942e-09
TRA2 vs A_n	31780	13574(42.7%)	6315(46.5%)	7259(53.5%)	5.628e-16
Chiifu-401 vs A_n	33043	15614(47.3%)	6811(43.6%)	8803(56.4%)	2.619e-57
A_r **vs** A_n		1987	740(37.2%)	1247(62.8%)	3.568e-30

[a] percentages of all expressed genes; [b] percentages of total differentially expressed genes.

A_r vs A_n represented DEGs shared by all *B. rapa* varieties versus A_n-subgenome of *B. napus*.

Supplementary Table 3. Differentially expressed genes (DEGs) between restituted *B. rapa* (RBR) and four natural *B. rapa*, as well as between RBR and A_n-subgenome of *B. napus*

	Total pairs	Total DEGs	Up-regulated	Down-regulated	P-value
BY vs RBR	31408	3142(10.0%)	1569(49.9%)	1573(50.1%)	0.957
TRA1 vs RBR	30729	10366(33.7%)	4824(46.5%)	5542(53.5%)	3.905e-12
TRA2 vs RBR	31096	13034(41.9%)	6070(46.6%)	6964(53.4%)	5.0791e-15
Chiifu vs RBR	32362	14184(43.8%)	6187(43.6%)	7997(56.4%)	3.0361e-52
A_r vs RBR		749	242(32.3%)	507(67.7%)	1.8473e-22
A_n vs RBR	31867	5995(18.8%)	3273(54.6%)	2722(45.4%)	1.174e-12

[a] percentages of all expressed genes; [b] percentages of total differentially expressed genes.

A_r vs RBR represented DEGs shared by all four *B. rapa* versus RBR.

A_n vs RBR represented DEGs between RBR and those in A_n subgenome of *B. napus* ZS11.

p-value: Binomial test p-value of the up and down regulated genes of the pairwise comparisons.

Supplementary Table 4. Differentially expressed genes between four natural *B. rapa* and three *B. oleracea*

	Total pairs	TotalDEGs[a]	Up-regulated[b]	Down-regulated[b]
BY_Cjielan	25309	10475(41.4%)	5590(53.4%)	4885(46.6%)
BY_ganlan	25669	10689(41.6%)	5896(55.2%)	4793(44.8%)
BY_TRC	25773	10666(41.4%)	5360(50.2%)	5306(49.7%)
chiifu_ganlan	25488	10184(39.9%)	4942(48.5%)	5242(51.5%)
Cjielan_chiifu	25377	9814(38.7%)	5212(53.1%)	4602(46.8%)
TRC_chiifu	26149	11956(45.7%)	6703(56.1%)	5253(43.9%)
Cjielan_TRA1	24289	9484(39.0%)	4645(48.9%)	4839(51.0%)
TRA1_ganlan	24933	11918(47.8%)	6341(53.2%)	5577(46.7%)
TRA1_TRC	24901	8194(32.9%)	3936(48.0%)	4258(51.9%)
Cjielan_TRA2	24617	10426(42.4%)	5179(49.7%)	5247(50.3%)
TRA2_ganlan	25142	12772(50.8%)	6762(52.9%)	6010(47.0%)
TRA2_TRC	25084	9195(36.7%)	4325(47.0%)	4870(52.9%)
A_r vs B_o		973	455(46.7%)	518(53.3%)

[a] percentages of all expressed genes; [b] percentages of total differentially expressed genes.

A_r vs B_o represented DEGs shared by all *B. rapa* and all *B. oleracea*.
